# Airborne Particulate Matter in Two Multi-Family Green Buildings: Concentrations and Effect of Ventilation and Occupant Behavior

**DOI:** 10.3390/ijerph13010144

**Published:** 2016-01-20

**Authors:** Allison P. Patton, Leonardo Calderon, Youyou Xiong, Zuocheng Wang, Jennifer Senick, MaryAnn Sorensen Allacci, Deborah Plotnik, Richard Wener, Clinton J. Andrews, Uta Krogmann, Gediminas Mainelis

**Affiliations:** 1 Environmental and Occupational Health Sciences Institute, Rutgers University, 170 Frelinghuysen Road, Piscataway, NJ 08854, USA; patton@eohsi.rutgers.edu; 2 Department of Environmental Sciences, Rutgers University, 14 College Farm Road, New Brunswick, NJ 08901, USA; calderon@envsci.rutgers.edu (L.C.); youyou@envsci.rutgers.edu (Y.X); jwang@iitri.org (Z.W.); krogmann@aesop.rutgers.edu (U.K.); 3 Edward J. Bloustein School of Planning and Public Policy, Rutgers University, 33 Livingston Ave., New Brunswick, NJ 08901, USA; jsenick@rci.rutgers.edu (J.S.); maryann.sa@rutgers.edu (M.S.A.); dplotnik@rci.rutgers.edu (D.P.); cja1@rutgers.edu (C.J.A.); 4 Department of Technology, Culture & Society, Polytechnic Institute of New York University, 6 MetroTech Center, Brooklyn, NY 11201, USA; rwener@nyu.edu

**Keywords:** particulate matter, green buildings, indoor air quality, ventilation, behavior

## Abstract

There are limited data on air quality parameters, including airborne particulate matter (PM) in residential green buildings, which are increasing in prevalence. Exposure to PM is associated with cardiovascular and pulmonary diseases, and since Americans spend almost 90% of their time indoors, residential exposures may substantially contribute to overall airborne PM exposure. Our objectives were to: (1) measure various PM fractions longitudinally in apartments in multi-family green buildings with natural (Building E) and mechanical (Building L) ventilation; (2) compare indoor and outdoor PM mass concentrations and their ratios (I/O) in these buildings, taking into account the effects of occupant behavior; and (3) evaluate the effect of green building designs and operations on indoor PM. We evaluated effects of ventilation, occupant behaviors, and overall building design on PM mass concentrations and I/O. Median PM_TOTAL_ was higher in Building E (56 µg/m^3^) than in Building L (37 µg/m^3^); I/O was higher in Building E (1.3–2.0) than in Building L (0.5–0.8) for all particle size fractions. Our data show that the building design and occupant behaviors that either produce or dilute indoor PM (e.g., ventilation systems, combustion sources, and window operation) are important factors affecting residents’ exposure to PM in residential green buildings.

## 1. Introduction

Government and private sectors have worked towards improving the environmental and human health performance of residential buildings [1,2]. Several programs (e.g., ENERGY STAR, LEED) exist to certify these buildings based on their energy efficiency and other environmental features [3,4,5,6]. These programs and their derivatives additionally provide guidance towards strategies with potential health benefits, including better air filtration, the use of low VOC–emitting materials, integrated pest management, and active living design [7]. In particular, design strategies such as improved ventilation of indoor spaces and filtration of incoming outdoor air could remove airborne particulate matter (PM), which is associated with cardiovascular and respiratory diseases [8,9,10,11,12,13,14].

Green buildings both in their design and operation may provide varying levels of health benefits and risks to their occupants [15,16,17]. However, the human health component of the buildings often is relegated to a few easy-to-score certification points, while certification programs and overlay standards with more emphasis on “healthy buildings” (e.g., the WELL Building Standard**^®^**, the National Healthy Housing Standard) are new to market [5,6]. Moreover, designing a building for maximum energy efficiency sometimes conflicts with achieving superior indoor air quality (IAQ), given that the latter may entail high air exchange rates via mechanical ventilation [15]. Building design and construction strategies and quality can vary widely, being influenced by the culture of the local area as well as the resources available. In addition, air quality might be heavily impacted by occupant behavior during building operation. Although indoor air quality was taken into account in all green building certifications considered in a recent review, and PM_10_ or PM_2.5_ control was mentioned in 16% of those certifications, only two certifications (LEED and HQE) specifically mentioned measurement of PM_2.5_ to confirm exposures [4,7,18].

Since most Americans spend almost 90% of their time indoors, it is important to characterize PM and other air pollutants in these settings—workplaces, schools and, especially, residences [19,20]. Previous studies have shown that indoor sources can contribute between 39% and 75% of residential PM_2.5_ (PM < 2.5 μm in aerodynamic diameter); PM_2.5_, a subset of PM, is of special interest because of observed relationships between PM_2.5_ exposures and cardiovascular and pulmonary health risks [21,22,23]. Indoor sources tend to be intermittent and depend on both occupant behaviors and building design; frying, grilling (with and without exhaust fans), burning candles that evaporate oil, and smoking can lead to peak PM concentrations of several hundred µg/m^3^ [24,25,26,27,28]. Higher concentrations of particles generated indoors are expected to occur in kitchens and bathrooms, and decrease with the use of exhaust ventilation systems or operation of window and central air conditioning systems [25,29]. Outdoor sources contribute to indoor concentrations through transmission in active (e.g., air handling units) and passive (e.g., open windows and leaks) ventilation systems. Infiltration of outdoor air can lead to high indoor concentrations of outdoor pollutants near industrial or traffic sources [30]. The relative contributions of indoor and outdoor sources depend on the magnitude of airflow, the level of filtration, and proximity of the strongest sources [26,31,32]. 

Because many factors including ventilation and occupant behavior (e.g., cleaning methods and their frequency) influence indoor air quality [33], it is not immediately apparent how ventilation system design and occupant behaviors affect PM in green residential buildings. Few studies have been conducted to measure PM in green residential buildings [34,35,36,37,38,39]. Three of these studies were conducted in retrofitted traditional buildings [36,37,38], two were in new LEED platinum buildings [35,39], and one was a new ENERGY STAR home [34]. All of these studies were conducted in multi-family green buildings or a mixture of single and multifamily residences. Those studies comparing traditional and green multifamily residences often had inconclusive results due to the small sample sizes [35,36,37,38]. More measurements are needed to characterize PM in residential green buildings, and to determine whether residents may be at increased risk of cardiovascular and respiratory diseases due to elevated PM levels [13,16,40,41,42]. 

To address the general lack of data on PM in green buildings, and particularly in multi-family residential green buildings, we measured PM in two residential high-rise green buildings constructed between 2000 and 2010 in the northeastern United States with differences in operation and construction. Our objectives were to: (1) measure particulate matter concentrations in apartments in these two buildings; (2) compare measurements made in the same building at different times, and between the two buildings, taking into account the effects of occupant behavior; and (3) evaluate the effect of green building ventilation system design on indoor PM by comparing measurements made in these green buildings to those reported in the literature for similar green and traditional buildings.

## 2. Experimental Section 

### 2.1. Buildings 

Monitoring was conducted in two residential high-rise green buildings completed between 2000 and 2010 in the northeastern United States (Table 1). Building management provided information about building features. Building E is an affordable ENERGY STAR housing development in which 88% of households had annual incomes of less than $20,000. This building was constructed with a natural air ventilation system (predominantly through windows) with local mechanically ventilated exhausts in the kitchens and the bathrooms (design flow = 30 ft^3^/min = 0.01 m^3^/s per exhaust vent). Ventilation code compliance had been waived and reduced rates employed to improve energy efficiency and performance. Approximately 73% of apartments (20% of visits in this study) had at least one window air conditioning (AC) unit drawing air from outside when operating. Building E is located about 100 meters from an elevated rail line and at the nexus of two side streets in an urban community known for a predominance of truck routes. Building L is a 27-floor LEED platinum certified luxury apartment building where 80% of households had annual incomes of more than $200,000. This building has a central ventilation system that supplies conditioned 100% outdoor air (*i.e.*, air pumped through intakes). Operable windows were rarely opened during this study, as recorded by the operator during his daily walk around the building and observed by the investigators. Flow rates of supply air into sampled apartments were 117–186 ft^3^/min (0.0552–0.0878 m^3^/s) in 2003 [39]. Each of the two outdoor air intakes (on the entrance floor and roof) had a MERV 7 filter followed by a MERV 14 filter. These filters were expected to remove >60 % of all particles, and ≥90% of particles <0.02 μm or >1 μm in diameter [43]. Local exhaust ventilation was installed in the kitchens and bathrooms (design flow rates = 120 ft^3^/min (0.057 m^3^/s) and 50 ft^3^/min (0.024 m^3^/s), respectively). Heating and cooling were provided separately from the supply air by fan-coil units fitted with MERV 11 filters. Building L was close to a diesel powered ferry station. Certain aspects of indoor air quality in Building L were previously described by Xiong *et al*. [39]. In both buildings, interior finishes (*i.e.*, paints, sealants, and caulks) were low VOC, and stoves were gas-fired with electronic ignition. Smoking was allowed in apartments but not in the common spaces of both buildings.

**Table 1:** Building descriptions.

Building	L	E
Designation	LEED platinum **^a^**	ENERGY STAR
Housing Type	Luxury	Affordable housing development (Economy)
Number of Floors	22	6 and 7 (two wings)
Design Exhaust Ventilation Rate	120 ft^3^/min (0.057 m^3^/s) in kitchens and 50 ft^3^/min (0.024 m^3^/s) in bathrooms	30 ft^3^/min (0.014 m^3^/s) each in kitchens and bathrooms
Supply Air Ventilation Rate	117–186 ft^3^/min (0.0552–0.0878 m^3^/s) [39]	None—naturally ventilated
Active Ventilation Systems	Conditioned 100% outside supply air; local bathroom and kitchen exhausts	Local kitchen and bathroom exhausts
Filtration	MERV 7 and 14 on air intakes; MERV 11 in units	No central filtration
Heating	Fan coil units	Baseboard heating
Stove Type	Gas	Gas
Air Conditioning	Central	Window units in ~73% of apartments

**^a^** The building qualified for three certifications: New York State Green Building Tax Credit (GBTC) Part 638, Tax Law Section 19; LEED v2.0 Gold certified in 2004; LEED-EB (Existing Buildings) Platinum in 2009.

### 2.2. Measurements 

#### 2.2.1. Measurement Campaigns

We visited the buildings in two separate but related research project campaigns between 27 April 2011 and 15 December 2013 (timeline presented in Supporting Information Figure S1). Measurements were made in Building E during both campaigns and in Building L during the second campaign only. The first campaign (C1) was conducted in Building E over three monitoring periods for a total of 55 apartment visits in C1-E: 13 July 2011–7 September 2011 (Phase I, Baseline measurement, *n* = 21), 24 October 2011–20 December 2011 (Phase II, post-intervention follow-up, *n* = 17), and 21 March 2012–22 May 2012 (Phase III, extended follow-up, *n* = 17), where “*n*” refers to the number of investigated apartments. The phases corresponded to the phases of a “green” cleaning behavioral intervention the results of which will be reported elsewhere. Participation in the study was offered to all building occupants via communications by management and the research team using flyers and meetings. Sixteen low-income households, largely from Latina and African American populations, voluntarily participated in all three phases of air quality measurements, 2 in two phases, and 3 in only one phase. 

The second campaign (C2) was conducted to compare indoor air quality parameters in Building E (same as in C1) and Building L. Measurements in Building E for Campaign 2 (C2-E) were made in 15 apartments from 22 August 2012 to 12 August 2013, approximately one year after completion of the first campaign, and participating apartments were visited 9–12 times each (mean = 11) for a total of 168 apartment visits in C2-E. Of the 15 apartments visited in C2-E, 10 had been visited in the first campaign. Campaign 2 measurements in 16 apartments of Building L were made from 27 April 2011–15 December 2011 and each apartment was visited between 2 and 9 times (mean = 7) for a total of 116 apartment visits in C2-L, temporally overlapping with both Phase I and Phase II of the first campaign.

#### 2.2.2. Measurement Procedures

Measurements were made for 45 min–1 h for the first campaign and 30–45 min for the second campaign, following a similar method to previous studies in green residences [35,36,38]. On each day, the first measurement was made on a table outside of the entrance floor (street level) of the building. Then, the same set of instruments was brought sequentially to 1–3 (C1) or 2–10 (C2, mean = 6) apartments between 09:00 and 17:00. Monitoring equipment was placed on a table 0.8 m–1.2 m off the ground in the living room (C2) or room where people spent most of their time during the day (C1, usually the living room). The order of apartment monitoring was determined by occupant availability, so was assumed to be relatively random. 

Airborne particle mass concentration fractions (PM_1_, PM_2.5_, PM_4_, PM_10_, and PM_TOTAL_) were measured with a DustTrak (DRX 8534, TSI, Shoreview, MN, USA). DustTraks have previously been used in similar studies comparing PM in different buildings and apartments [36,38]. We did not use a correction factor for comparability with other monitors because all measurements in this study were made with the same DustTrak and we were most interested in relative concentrations [44,45]. The DustTrak DRX is a photometer, where a laser diode illuminates a sample stream and reflected light is directed onto a photodetector by a mirror [46]. The photometric voltage output by the DustTrak was converted to size-segregated mass concentrations of PM based on calibration with Arizona road dust [46]. The DustTrak was calibrated and used according to manufacturer’s recommendation. The monitor was within a year of manufacturer calibration for all parts of the two measurement campaigns, and a zero control was performed at the start of each measurement day. Measurements had good correlation with gravimetric measurements (unpublished data). In addition, measurements were inspected for unreasonable values in post-processing, and all values were retained in the dataset because no outliers occurred when there was no reasonable explanation (e.g., visible PM source).

Temperature and relative humidity were measured with an IAQ meter (model 7545, TSI Inc., Shoreview, MN, USA) during all PM measurements. Since PM varies seasonally, temperature and humidity were used to evaluate seasonal effects.

In addition, the local exhaust ventilation rates in a subset (*n* = 14) of the Building E apartments were determined by air velocity measurements. Air velocity was measured with a VelociCalc (model 9535-A, TSI, Inc., Shoreview, MN, USA) in each of four quadrants of each exhaust vent for 45 s, averaged, and multiplied by the cross-sectional vent area (0.034 m^2^ in kitchens and 0.018 m^2^ in bathrooms) to arrive at flow rates. This type of measurement is usually assumed to be accurate within about 10%, although methods to determine uncertainty due to duct geometry have not been reported [47].

#### 2.2.3. Determination of Occupant Behaviors

In both campaigns, the investigators recorded occupant behaviors during measurements. For information on air exchange, it was noted whether windows were open or closed or air conditioners were running. Combustion-producing behaviors (*i.e.*, smoking, cooking, incense or candle burning) were noted and each apartment visit’s combustion status was classified as yes (active combustion), maybe (signs of recent combustion), or no (no active combustion or signs of recent combustion). Signs of recent combustion included the presence of cigarettes, cooking, candles, and incense.

Self-reported occupant behaviors including whether a smoker lived in the apartment, window air conditioners were used, and other behaviors were collected on questionnaires at the start of Campaign 1. No formal interviews were conducted during the second campaign. 

#### 2.2.4. Statistical Analysis

Analysis of PM concentrations in the two buildings was done on both a mass concentration and indoor/outdoor mass concentration ratio (I/O) basis for all measured particle size fractions. I/O ratios were calculated to summarize the overall effects of indoor and outdoor sources, filtration, and air supply on presence of PM in indoor air relative to ambient air. For each size fraction, the following summary statistics were calculated for PM mass concentration and indoor/outdoor ratio: the number of available and missing measurements, minimum, 25th percentile, median, mean, 75th percentile, and maximum. The distributions of concentrations also were compared using both box plots and histograms.

Because the measurements were skewed (consistent with lognormal distributions), nonparametric statistical tests were used to test the effects of ambient conditions, building features, and occupant behaviors on airborne PM concentrations. Differences between groups (e.g., apartments with or without combustion during sampling) were evaluated on a categorical basis using Kruskal-Wallis nonparametric multiple comparisons test. Effects of continuous variables were evaluated by log-linear regression. Trends in statistical significance as a function of particle size fractions were also explored. Statistical tests were done independently for C1-E, C2-E, and C2-L, and also for the pooled data from Building E (C1-E and C2-E). Because the outdoor PM concentrations and operational conditions were different in the two buildings, data were not pooled between the buildings. The null hypothesis of no difference between groups was rejected for *p* < 0.05. All statistical tests were conducted in R 3.1.0 [48]. 

### 2.3. Meta-Analysis Comparison with Literature

Results obtained from this study were compared to other studies in residential green buildings using PM_2.5_ as the main metric. Similar studies reporting PM_2.5_ mass concentrations in green, residential, and high-rise buildings were identified by searching PubMed for “green building indoor air” and “low energy indoor air”. For each study, the following data were extracted: study name, year published, number of residential units, measurement duration, measurement instrument, housing occupants (e.g., low-income, seniors), housing type (single-family or multi-family), healthy housing category (e.g., green, traditional), investigated room (living room or bedroom), ventilation type, and all available PM_2.5_ concentration statistics. For studies that did not report the geometric mean and geometric standard deviation, these statistics were estimated assuming a lognormal distribution of PM_2.5_ mass concentrations. Differences in geometric mean between green and traditional buildings in the same study were summarized in a forest plot and tested for statistical significance using log-scale fixed-effects meta-analysis in the meta.summaries function of the rmeta package in R [49]. In addition, the geometric mean, mean and 95% confidence intervals for measurements in green buildings were summarized for comparison to this study.

### 2.4. IRB Statement

All subjects gave their informed consent for inclusion before they participated in the study. The study was conducted in accordance with the Declaration of Helsinki, and the protocol was approved by the Institutional Review Board of Rutgers University (protocols # 10-M713 and E15-153).

## 3. Results and Discussion

### 3.1. Summary Statistics and Comparison

**Figure 1:**
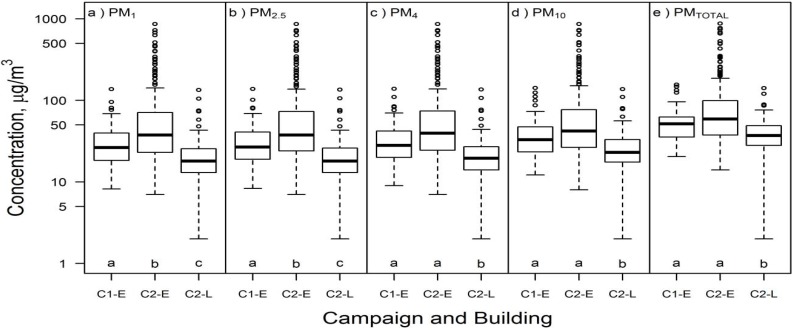
Airborne particulate mass concentration (µg/m^3^) in Campaign 1 Building E (C1-E; *n* = 55), Campaign 2 Building E (C2-E; *n* = 168), and Campaign 2 Building L (C2-L; *n* = 116) for (**a**) PM_1_; (**b**) PM_2.5_; (**c**) PM_4_; (**d**) PM_10_; and (**e**) PM_TOTAL_. Different letters within the same box represent statistically significant (*p* < 0.05) groups under the Kruskal-Wallis multiple comparisons test.

Airborne particulate matter concentrations of all size fractions reported by the DustTrak are summarized in Figure 1, Figure S2, and Table S1. Median PM_TOTAL_ in Building E was 56 µg/m^3^ for pooled data, 52 µg/m^3^ for C1-E only, and 59 µg/m^3^ for C2-E only. According to the Kruskal-Wallis multiple comparisons test, there was no statistically significant difference in PM_4_, PM_10_ and PM_TOTAL_ between the two Building E campaigns; however, C2-E concentrations were more variable (interquartile range = 38–97 µg/m^3^ during C2-E *vs.* 36–63 µg/m^3^ during C1-E) and sometimes much higher than C1-E concentrations. PM_TOTAL_ median in Building L was 37 µg/m^3^; it was significantly lower than in Building E (*p* < 0.05). A significant fraction of the measured PM in C1-E, C2-E, and C2-L was < 1 μm in diameter: the PM_1_ interquartile range was 16–45 µg/m^3^, or 46%–75% (median 61%) of the total measured PM mass concentration.

**Figure 2:**
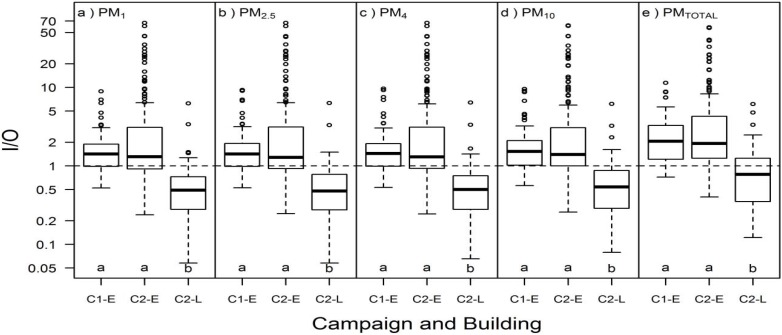
I/O in Campaign 1 Building E (C1-E; *n* = 55), Campaign 2 Building E (C2-E; *n* = 168), and Campaign 2 Building L (C2-L; n = 116) for (**a**) PM_1_; (**b**) PM_2.5_; (**c**) PM_4_; (**d**) PM_10_; and (**e**) PM_TOTAL_. Different letters within the same box represent statistically significant (*p* < 0.05) groups under the Kruskal-Wallis multiple comparisons test. Dashed lines for equal indoor and outdoor concentrations (I/O = 1) are included for reference.

Indoor/outdoor (I/O) ratios in Building E ranged from 0.24 to 66, while in Building L they ranged from 0.06 to 6.4 (Figure 2; Figure S3, Table S1). Median I/O ratios in Building E were 1.3–1.4 for particle sizes smaller than 10 µm and 2.0 for PM_TOTAL_. All 17 cases with PM_2.5_ I/O > 10 were in C2-E and were registered in 7 unique apartments; although detailed occupant behavior data were not available for C2-E, this observation of high I/O in the same apartments over multiple visits clearly suggests substantial particle-generating behaviors by the residents. In Building L, median I/O ratios for PM_1_, PM_2.5_, PM_4_, and PM_10_ were approximately 0.5 and median I/O ratio for PM_TOTAL_ was 0.8. Of all cases with PM_2.5_ I/O < 0.5, 49 were from C2-L and two were from a single day of relatively high outdoor PM_2.5_ (58 μg/m^3^) in C2-E. Both lower indoor and higher outdoor concentrations (data not shown) contributed to the lower I/O in Building L compared to Building E (*p* < 0.05 for all particle sizes).

Considering only apartments with the most similar occupant behaviors in Building E and Building L (*i.e.*, closed windows and no combustion sources) increased the similarity of concentrations and indoor/outdoor ratios between the buildings, but did not account for all of the differences. For this subset of the measurements, there was little statistical significance in particulate mass concentration differences between the buildings (Figure S4). The only statistical difference was for PM_2.5_, which had medians of 25 μg/m^3^ in Building E (mean = 25 μg/m^3^) and 18 μg/m^3^ in Building L (mean = 22 μg/m^3^). In this subset of data, the indoor/outdoor ratios for Building E trended towards 1 (Figure S5). However, indoor/outdoor ratios in Building E did not go below 1 as measured in Building L.

### 3.2. Effects Related to Occupant Behaviors

Occupant behaviors that affected PM concentrations in Building E included use of combustion sources (stoves, incense, and to a lesser effect, smoking), the operation of air conditioning units, and window opening. During measurements in Building L, tenants usually were not home, and no one smoked or cooked in the apartments.

#### 3.2.1. Combustion Sources in Building E

**Figure 3:**
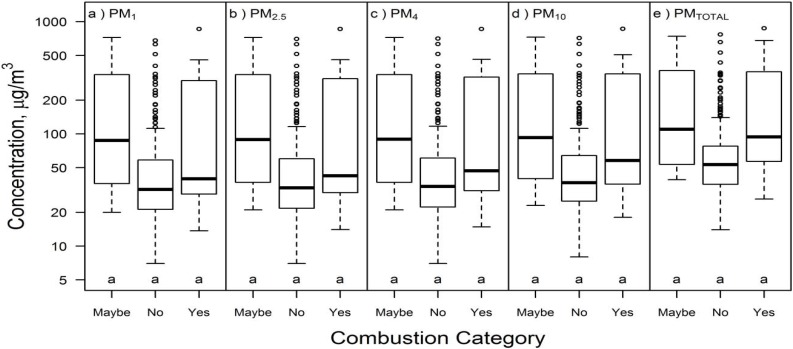
Airborne PM mass concentration (μg/m^3^) in Building E (both campaigns) for (**a**) PM_1_; (**b**) PM_2.5_, (**c**) PM_4_; (**d**) PM_10_; and (**e**) PM_TOTAL_ based on investigator-observed combustion activities during measurements: yes = combustion source during sampling (*n* = 12), maybe = investigators observed incense or cigarettes (*n* = 8), and no = no investigator observations of combustion or likely combustion sources (*n* = 203). Different letters within the same box represent statistically significant (*p* < 0.05) groups under the Kruskal-Wallis multiple comparisons test.

**Figure 4:**
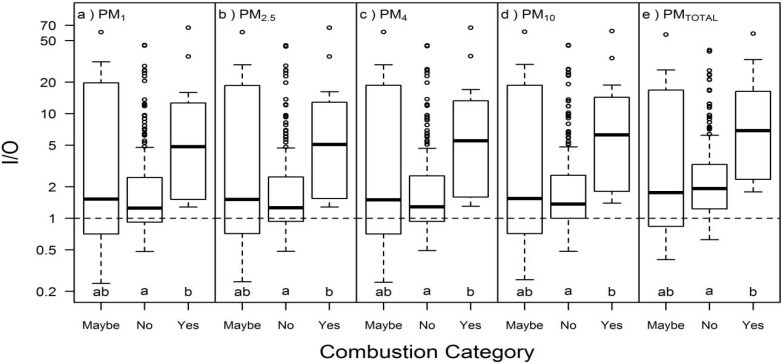
Boxplots of I/O in Building E (both campaigns) for (**a**) PM_1_, (**b**) PM_2.5_, (**c**) PM_4_, (**d**) PM_10_, and (**e**) PM_TOTAL_ based on investigator-observed combustion activities during measurements: yes = combustion source during sampling (*n* = 12), maybe = investigators observed incense or cigarettes (*n* = 8), and no = no investigator observations of combustion or likely combustion sources (*n* = 203). Different letters within the same box represent statistically significant (*p* < 0.05) groups under the Kruskal-Wallis multiple comparisons test. Dashed lines for equal indoor and outdoor concentrations (I/O = 1) are included for reference.

PM mass concentration (all size fractions) in Building E was higher during use of combustion sources during Campaign 2, but not during Campaign 1 (Table 2). The median PM concentration for all particle sizes in Building E was lower in apartments without active combustion sources (e.g., PM_TOTAL_ median = 53 μg/m^3^) than in apartments with active combustion sources (e.g., PM_TOTAL_ median = 94 μg/m^3^; Figure 3). Similarly, the I/O ratio of all PM size fractions was higher in apartments with active combustion sources than apartments without active combustion sources (Figure 4).

Surprisingly, the PM mass concentrations and indoor-outdoor ratios did not appear to be affected by having a smoker live in the apartment. Although PM mass concentrations were 4%–9% lower in smoking apartments in C1-E than in non-smoking apartments (e.g., PM_2.5_ median = 25 µg/m^3^ for smoking and 27 µg/m^3^ for not smoking), this difference was not statistically significant. Apartments with occupants who smoked also had similar PM I/O to non-smoking apartments (*p* = 0.33–0.55; Table 2). Most of the upper extreme PM concentrations were in apartments where investigators observed evidence of current or recent smoking, cooking, or candle/incense burning (data not shown), so the smoking question on the survey may have insufficiently characterized the presence of smoking in the apartments.

**Table 2:** Kruskal-Wallis tests for particulate mass (µg/m^3^) and indoor/outdoor ratio by apartment smoking, air conditioning, and combustion.

Site	Pollutant	Mass Concentration, µg/m^3^			Indoor/Outdoor Ratio		
		Yes ^a^	No ^a^	*p*	Yes ^a^	No ^a^	*p*
Smoker in apartment **^b^**							
C1-E	PM_1_	25 (18, 41)	26 (20, 40)	0.972	1.4 (0.9, 2.0)	1.5 (1.1, 1.8)	0.554
	PM_2.5_	25 (18, 41)	27 (20, 41)	0.917	1.4 (0.9, 2.0)	1.5 (1.1, 1.9)	0.508
	PM_4_	26 (19, 42)	28 (21, 42)	0.931	1.4 (0.9, 1.9)	1.5 (1.2, 1.9)	0.465
	PM_10_	31 (21, 48)	34 (26, 47)	0.889	1.4 (0.9, 2.1)	1.7 (1.2, 2.1)	0.394
	PM_TOTAL_	51 (35, 63)	50 (38, 60)	0.821	2.0 (1.1, 2.7)	2.2 (1.4, 3.6)	0.330
Operating window AC (outdoor temperature > 70 °F (21.1 °C)) **^c^**							
C1-E	PM_1_	24 (17, 49)	27 (22, 38)	0.930	1.1 (1.0, 1.6)	1.6 (1.5, 2.9)	0.268
	PM_2.5_	25 (17, 49)	28 (22, 39)	0.930	1.1 (1.0, 1.6)	1.6 (1.5, 2.9)	0.246
	PM_4_	26 (18, 50)	29 (23, 42)	0.881	1.1 (1.0, 1.6)	1.6 (1.6, 3.0)	0.246
	PM_10_	28 (20, 52)	37 (29, 49)	0.720	1.1 (1.0, 1.8)	1.9 (1.7, 3.5)	0.171
	PM_TOTAL_	42 (29, 58)	62 (57, 67)	0.192	1.5 (1.1, 2.2)	3.0 (2.1, 6.4)	0.052
C2-E	PM_1_	47 (39, 61)	42 (33, 60)	0.713	1.0 (0.9, 1.3)	1.2 (0.8, 1.8)	0.670
	PM_2.5_	47 (39, 61)	43 (34, 61)	0.729	1.1 (0.9, 1.3)	1.2 (0.8, 1.8)	0.673
	PM_4_	48 (39, 62)	43 (34, 62)	0.763	1.1 (0.9, 1.3)	1.2 (0.8, 1.7)	0.724
	PM_10_	48 (40, 62)	47 (37, 63)	0.853	1.1 (0.9, 1.2)	1.2 (0.8, 1.7)	0.717
	PM_TOTAL_	55 (46, 69)	65 (50, 82)	0.736	1.1 (0.9, 1.4)	1.6 (1.0, 2.3)	0.621
E	PM_1_	41 (21, 53)	40 (32, 56)	0.555	1.1 (0.9, 1.5)	1.2 (0.9, 1.9)	0.547
	PM_2.5_	42 (22, 53)	41 (33, 56)	0.529	1.1 (0.9, 1.5)	1.2 (0.8, 1.8)	0.547
	PM_4_	42 (22, 54)	42 (33, 58)	0.513	1.1 (1.0, 1.6)	1.2 (0.8, 1.9)	0.611
	PM_10_	44 (23, 55)	46 (35, 62)	0.393	1.1 (1.0, 1.8)	1.3 (0.8, 2.0)	0.606
	PM_TOTAL_	53 (32, 62)	64 (51, 81)	0.053	1.4 (1.1, 2.1)	1.6 (1.1, 2.6)	0.551
Combustion **^d^**							
C1-E	PM_1_	31 (24, 40)	26 (17, 40)	0.419	2.2 (1.5, 4.8)	1.3 (1, 1.7)	0.025
	PM_2.5_	32 (25, 42)	26 (18, 40)	0.363	2.1 (1.5, 5.1)	1.3 (1, 1.7)	0.020
	PM_4_	34 (26, 47)	27 (19, 41)	0.325	2 (1.6, 5.5)	1.3 (1, 1.7)	0.016
	PM_10_	43 (29, 58)	33 (24, 45)	0.350	2 (1.8, 6.3)	1.4 (1, 2.1)	0.026
	PM_TOTAL_	62 (39, 81)	50 (36, 61)	0.495	2.6 (2, 6.9)	1.9 (1.1, 2.9)	0.046
C2-E	PM_1_	398 (198, 457)	36 (23, 68)	0.005	15.9 (9.4, 35.2)	1.2 (0.9, 2.8)	0.016
	PM_2.5_	422 (199, 458)	37 (24, 70)	0.004	16.2 (9.5, 35.2)	1.3 (0.9, 2.8)	0.016
	PM_4_	442 (200, 462)	37 (24, 70)	0.004	17 (9.5, 35.5)	1.3 (0.9, 2.8)	0.014
	PM_10_	476 (208, 507)	42 (26, 73)	0.004	18.8 (9.9, 34)	1.4 (1, 2.8)	0.013
	PM_TOTAL_	493 (223, 680)	58 (36, 88)	0.001	21.2 (10.1, 32.9)	1.9 (1.2, 3.6)	0.007
E	PM_1_	40 (29, 248)	33 (22, 61)	0.025	4.8 (1.5, 11.1)	1.3 (0.9, 2.5)	0.003
	PM_2.5_	42 (30, 255)	33 (22, 62)	0.020	5.1 (1.6, 11.2)	1.3 (0.9, 2.5)	0.002
	PM_4_	47 (31, 260)	34 (23, 63)	0.016	5.5 (1.6, 11.4)	1.3 (0.9, 2.6)	0.002
	PM_10_	58 (36, 275)	39 (26, 66)	0.013	6.3 (1.9, 12.1)	1.4 (1, 2.6)	0.002
	PM_TOTAL_	94 (59, 290)	54 (36, 81)	0.009	6.9 (2.5, 13.9)	1.9 (1.2, 3.3)	0.003

**^a^** Mass concentration and indoor/outdoor ratio are presented as median (25th percentile, 75th percentile). **^b^** Whether a smoker lived in the apartment was defined as the answer to the survey question. There were 30 apartments with smokers and 24 without smokers in C1-E. **^c^** Window AC use analyses included Building E only because there were no window AC units in Building L. Window air conditioners were operating in 11 C1-E apartment visits and 9 C2-E apartment visits. There were no operating window air conditioners during 11 C1-E apartment visits and 146 C2-E apartment visits. **^d^** Combustion analyses included Building E only because there was no combustion during measurements in Building L. Active combustion during measurements took place in 7 (C1-E) and 5 (C2-E) apartments in each campaign, for a total of 12 Building E apartments with active combustion sources. The remaining 48 (C1-E) and 163 (C2-E) apartment visits in Building E did not have active combustion).

#### 3.2.2. Air Conditioning and Window Opening in Building E

On hot monitoring days (>21.1 °C (70 °F)) when some apartments had running window air conditioners (AC), the effect of air conditioning on particulate mass concentration in Building E trended towards a significant PM decrease with increasing particle sizes (Table 2). Twenty measurement sessions were conducted when window or portable air conditioning units were being used, 11 of which were during C1-E and 9 of which were in C2-E. Median PM_TOTAL_ mass was lower in units with running air conditioners than in units without running air conditioners although the difference was small for all practical purposes (1.4 µg/m^3^ *vs.* 1.6 µg/m^3^, *p* = 0.02). The effect of AC on I/O was not significant (*p* = 0.37–0.75; Table 2). Air conditioning was not evaluated as a factor in Building L because all apartments were equipped with centralized air conditioning and did not have window AC units.

**Figure 5:**
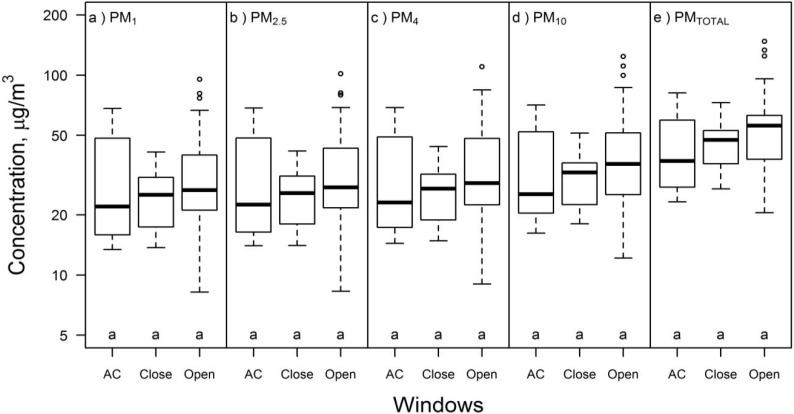
Boxplots of PM mass concentration (μg/m^3^) in Building E (both campaigns) for (**a**) PM_1_, (**b**) PM_2.5_; (**c**) PM_4_; (**d**) PM_10_; and (**e**) PM_TOTAL_ based on window opening. Categories are AC on (AC, windows presumably closed, *n* = 10), windows closed (Close, *n* = 16), and windows open (Open, *n* = 25). Different letters within the same box represent statistically significant (*p* < 0.05) groups under the Kruskal-Wallis multiple comparisons test.

**Figure 6:**
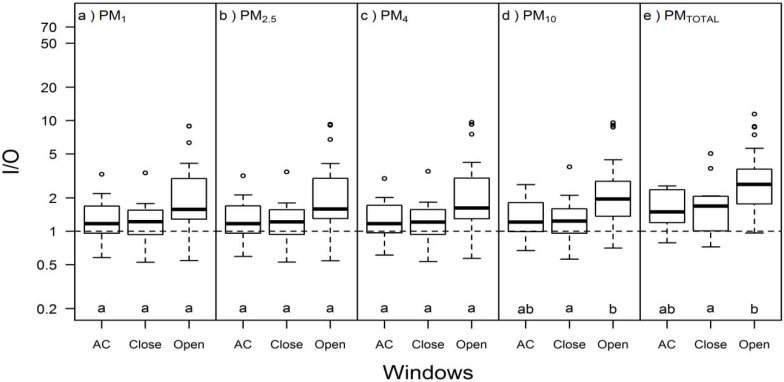
Boxplots of I/O in Building E (both campaigns) for (**a**) PM_1_; (**b**) PM_2.5_; (**c**) PM_4_; (**d**) PM_10_; and (**e**) PM_TOTAL_ based on window opening. Categories are AC on (AC, windows presumably closed, *n* = 10), windows closed (Close, *n* = 16), and windows open (Open, *n* = 25). Different letters within the same box represent statistically significant (*p* < 0.05) groups under the Kruskal-Wallis multiple comparisons test. Dashed lines for equal indoor and outdoor concentrations (I/O = 1) are included for reference.

Both PM mass concentrations and their I/O ratios in Building E were lowest when the windows were closed or the air conditioning was on (Figure 5; Figure 6). The median absolute differences in PM_TOTAL_ mass concentration and I/O for apartments with open windows relative to those with running air conditioning were 19 μg/m^3^ and 1.0, respectively, although these differences were not statistically significant due to large within-apartment variation. In Building E, closed windows (recorded as windows closed or AC on) were associated with lower PM_TOTAL_ mass concentration (Kruskal-Wallis *p* = 0.016) and PM_TOTAL_ I/O ratio (Kruskal-Wallis *p* = 0.005) than open windows. Possible explanations for these results are that windows were open to air out the room when PM concentrations were high or that the draft due to open windows resuspended deposited dust thus increasing airborne PM concentrations.

### 3.3. Effects Related to Building Design

#### 3.3.1. Exhaust Ventilation in Building E

Total local exhaust ventilation rates measured in 14 Building E apartment kitchens and bathrooms varied from apartment to apartment (mean: 63.6 ft^3^/min = 0.0300 m^3^/s, range: 0.6–111.0 ft^3^/min = 0.0003–0.0524 m^3^/s). The active ventilation rate at individual exhaust vents was typically higher in the bathroom (mean: 38.9 ft^3^/min = 0.0184 m^3^/s) than in the kitchen (mean: 24.7 ft^3^/min = 0.0117 m^3^/s). Measurements in the four quadrants of each exhaust vent tended to be uniform, with mean coefficients of variation of 3% (range = 0%–24%). Since the exhaust ventilation rates were not determined at the time of the measurements, temperature, wind speed, and positioning of doors and windows may have changed, leading to different exhaust ventilation rates during measurements compared to those reported here [48]. 

The effects of total exhaust ventilation on PM mass concentration and I/O in Building E were < 1% (*p* ≥ 0.05; Table S2). While the apartment with the highest PM mass concentrations in C2-E had the lowest ventilation rate (0.6 ft^3^/min = 0.0300 m^3^/s), apartments with the highest and lowest exhaust ventilation rates generally did not have extreme PM or I/O values. In addition, incense was in use during multiple sampling sessions in this apartment, so both combustion sources and limited ventilation likely contributed to the high PM mass concentrations. Although local exhaust ventilation was not measured in the C1-E apartment with the highest PM_TOTAL_ mass concentration, the apartment with the second highest airborne PM mass concentration had relatively high ventilation overall (74.5 ft^3^/min = 0.0352 m^3^/s), and lower than average ventilation in the kitchen (25.0 ft^3^/min = 0.0118 m^3^/s).

#### 3.3.2. Environmental Conditions

Environmental conditions were more consistent in Building L than in Building E due to mechanical air supply and conditioning systems. The lowest temperatures in both buildings were about 20 °C (68 °F), and the maximum recorded temperatures in any apartment were higher in Building E (31.7 °C = 89 °F) than in Building L (26.7 °C = 80 °F; Table S3). On average, outdoor temperatures were lower during sampling in C2-E (mean = 14.4 °C = 58 °F) than in C1-E (mean: 20.0 °C = 68 °F) or C2-L (mean: 21.1 °C = 70 °F). Relative humidity tended to be higher with less variability in Building L (mean = 53%, interquartile range = 50%–59%) than in Building E (combined campaigns mean = 38%, interquartile range = 24%–52%; Table S3). As per American Society of Heating, Refrigerating and Air-Conditioning Engineers (ASHRAE) recommended comfort zones, the air in Building E was often too hot and humid in summer and too dry in winter, and Building L was comfortable on nearly all days [50].

I/O ratio of PM mass concentration, but not PM mass concentration itself, was associated with differences in indoor and outdoor temperatures (Table S4). PM mass concentrations were not associated with indoor temperature, outdoor temperature, or I/O of temperature in C1-E, C2-E, or C2-L (*p* > 0.23 for all). In both C2-E and C2-L, the I/O ratio of different PM size fractions increased by 0.7–3.5 times per unit increase in I/O ratio of temperature and decreased by 3%–6% per 1 °C increase in outdoor temperatures. I/O of PM in C2-E was associated with 7%–9% borderline statistically significant (*p* = 0.01–0.08) decrease per 1 °C indoor temperature increase. There was no association of I/O of PM and I/O ratio of temperature or outdoor temperature in C1-E or C2-L.

PM mass concentration and I/O had mixed associations with relative humidity (Table S5). PM mass concentration increased by 0.3%–1.0% per 1% increase in indoor relative humidity; this effect was statistically significant in C2-E and C2-L, but not C1-E, possibly due to the smaller number of visits to each apartment in Campaign 1. PM mass concentration in C2-L increased by 65%–87% per 1% increase in indoor/outdoor ratio of relative humidity, an effect driven by a small number of samples in apartments with much higher relative humidity than outdoor conditions. I/O of PM decreased with increasing indoor (21%–24% per 1% increase in RH) and outdoor (<2% per 1% increase in RH) relative humidity in Building E during Campaign 2, but not in Building E during Campaign 1 or in Building L. In C2-L, a 1% increase in the indoor/outdoor ratio of RH was associated with 28%–79% increases in PM I/O.

#### 3.3.3. Building Floor

The effect of building floor on PM concentration was evaluated to determine whether systematic differences in PM occurred at different locations within the buildings, for example due to vertical gradients in ambient concentrations. In both Buildings E and L, PM concentrations were statistically different on at least one floor according to the Kruskal-Wallis multiple comparisons test, with lower *p*-values for larger particle sizes (Table S6). During Campaign 1, the Building E floor effect was dominated by high PM mass concentrations in the apartments on floor 3 (median PM_TOTAL_ = 74 μg/m^3^). This floor had combustion due to cooking with oil in half the apartments, while no other floor had cooking with oil, suggesting that the higher concentrations on this floor were dominated by occupant behaviors and not building characteristics. The effect of increasing building floor for Building E measurements from both campaigns resulted in 4%–8% decreases of all PM subsets for each floor (*p* = 0.04–0.06), although these decreases were not statistically significant in individual campaigns (*p* = 0.11–0.19; Table S7). PM subsets in Building L decreased by 1%–2% per floor, with statistical significance only for the PM_TOTAL_ (*p* = 0.03 *vs.* *p* = 0.19–0.32).

The effects of floor were more pronounced for I/O ratios. While the differences among floors by multiple comparison test (Table S6) and the log-linear regression by floor number (Table S7) both had mixed levels of statistical significance, the log-linear regression consistently estimated decreases in I/O for increasing floor height. Overall, PM mass concentration decreased by 6% per floor in building E (*p* = 0.06–0.09) and 1%–2% per floor in building L (*p* = 0.12–0.31).

### 3.4. Comparison to Other Green and Conventional Residential Buildings

The PubMed search for PM_2.5_ studies conducted in green buildings resulted in 204 hits, of which 5 studies reported PM_2.5_ measurements in a total of 8 different green residential buildings (see the excel file “Multi-study comparison of PM_2.5__SUBMISSION_SI”). Four of the studies included comparisons to traditional “non-green” buildings [35,36,37,38]. All of the studies were conducted in low-income multifamily residences, except for Offermann [34], who measured PM_2.5_ in single-family homes and did not describe the occupants. All published studies were in non-smoking buildings, except for Frey *et al.* (2014), who measured PM_2.5_ in senior residences that allowed smoking [36]. The green building ventilation systems were highly heterogeneous and included mechanical ventilation [37], mechanical ventilation with local exhaust fans [35], a mixture of natural and mechanical ventilation [34], and local exhaust fans [36,38]. One study included a building with energy recovery ventilation [38]. The green features were mostly energy improvements, but some new buildings also had low-emission construction materials.

**Figure 7:**
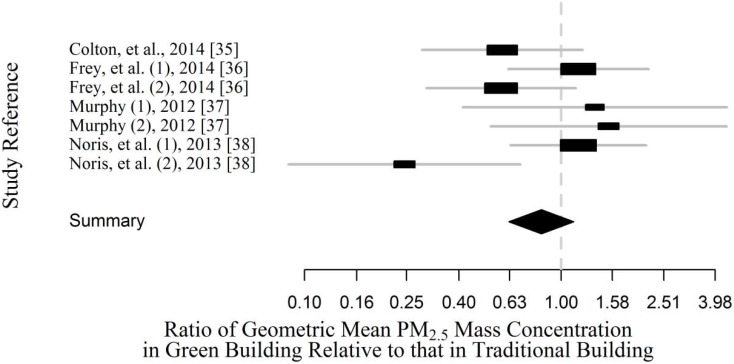
Fixed-effects meta-analysis of studies comparing PM_2.5_ in green and traditional multi-family residences. Study labels are author (building if multiple green buildings in one study), year [reference]. The summary statistic for each study is the geometric mean in green buildings divided by the geometric mean in traditional buildings. Geometric mean (boxes) and geometric standard deviation (lines) are shown for individual studies. The diamond summarizes the ratio of geometric means (diamond center) and 95% confidence interval (diamond width) for the combined studies.

In those studies that reported measurements for both low energy green (as defined by the original study authors) and traditional buildings, green buildings had similar or lower PM_2.5_ concentrations compared to traditional buildings (Figure 7). Overall, the geometric mean PM_2.5_ concentrations in green buildings as predicted by fixed effects models were 84% (95% CI: 63%, 110%) of the concentrations in the traditional buildings. These results are likely to change as the number of studies in green residences increases. If the building with the largest difference from the traditional building baseline (Noris *et al*. (2), 2013 [38]) were excluded, the PM_2.5_ concentrations in green buildings would be 95% (95% CI: 69%, 125%) of the concentrations in traditional buildings. Both fixed and mixed effects models were built and had essentially the same results.

**Figure 8:**
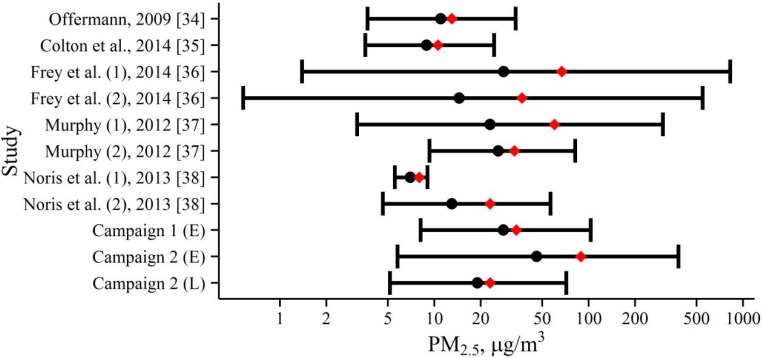
Summary of PM_2.5_ concentrations measured in multi-family green buildings. Geometric mean (black circles), mean (red diamonds) and range containing 95 percent of the measurements (whiskers) are shown. Study labels are author (building if multiple green buildings in one study), year [reference]. Measurement methods used included 24-hour gravimetric filter samples (Offerman [34], Murphy [37]), DustTraks (Frey *et al*. [36], Noris *et al*. [38], this study), and 6–10 day integrated filter samples (Colton [35]).

There was high variability of PM_2.5_ mass concentrations within and between the green buildings (Figure 8), with concentrations typically higher in buildings where smoking occurred. Our data in Building L (geometric mean (GM) = 18 µg/m^3^) were similar to those in other green buildings, while pooled measurements in Building E (GM = 28 µg/m^3^) were higher than previously reported measurements of PM_2.5_ mass concentrations. The most comparable multi-family green residences to Buildings E and L in terms of building age were relatively new green residences in Boston [35] and Chicago [37], and a low-energy senior residence in Arizona [36]. Of these studies, the lowest geometric mean PM_2.5_ was reported by Colton *et al*. (2.2 µg/m^3^ *vs.* 2.9 µg/m^3^–3.8 µg/m^3^ in the other studies), who also had the longest monitoring time (6–10 days) and used a filter-based measurement method (HPEM) [35]. The range and central tendencies of our data were similar to those reported by Frey *et al*. and higher than those reported by Norris *et al*., both of whom also used DustTraks for their PM measurements [36,38]. Regardless of the different measurement methods, occupancy, and times of measurement used in different studies, each building had a wide range of PM_2.5_ concentrations, often at relatively high levels.

## 4. Discussion of Results

### 4.1. Comparison of Two Green Buildings

The two green residential high-rise buildings that were visited in this study had differences in ventilation system design, construction, and operation, and different indoor PM concentrations. Building-level differences included both ventilation systems (conditioned, filtered 100% outside supply air in Building L vs natural ventilation and local exhaust ventilation in building E) and typical participant behaviors (e.g., occupants did not perform combustion-generating behaviors during sampling in Building L, while evidence of such behavior was apparent in Building E). The effects of building factors, occupant behaviors, and environmental conditions on PM mass concentrations and I/O ratios are summarized in Table 3. An interesting point was that mean I/O for PM_2.5_ was >1 for Building E and <1 for Building L (Figure 2, Table S1, Figure S3). Therefore, Building L had a net removal of PM_2.5_ relative to ambient concentrations while Building E was enriched in PM_2.5_ relative to ambient concentrations. The low I/O in Building L was likely due to the two sets of filters for outside air (supply air and fan-coil units) as well as limited indoor combustion sources. In contrast, the high I/O in Building E was likely due to indoor sources introduced by occupant behaviors, and possible also due to the low exhaust ventilation rates trapping both indoor and outdoor PM inside of the apartments. High PM concentrations and I/O ratios in Building E were consistent with complaints by most participants about dust in the apartments. Since the outdoor measurements were performed only once each day and close to the time of day with peak concentrations in this region [51], I/O ratios may have been underestimated for apartments that were visited later in the day, especially for the naturally ventilated building.

**Table 3:** Summary of the effects of building factors, occupant behaviors, and environmental conditions on PM mass concentration and I/O ratios.

Factor	Effect on PM Mass Concentration	Effect on PM I/O Ratios
Building factors		
Building	All size fractions lower in Building L (e.g., 19 μg/m^3^ difference in median PM_TOTAL_)	Building E: >1; Building L: <1
Change per increasing floor number	Building E: 4%–8% decrease; Building L: 1%–2% decrease	Building E: 6% decrease; Building L: 1%–2% decrease
Occupant behaviors in building E		
Combustion behaviors during measurements	C1-E: Increased peaks with median unchanged; C2-E: ~10 times higher than without combustion	C1-E: Increased peaks with median unchanged; C2-E: ~13 times higher than without combustion
Smoker lives in apartment	C1-E: None	C1-E: None
Operating window AC	C1-E and C2-E: Same as with closed windows	C1-E and C2-E: Same as with closed windows
Open windows	Building E pooled: Non-statistically significant increase of <20 μg/m^3^	Building E pooled: ~1 unit (~40%) increase with borderline statistical significance
Environmental conditions (indoor, outdoor, and I/O)		
Temperature	None	C1-E: None; C2-E and C2-L: Increased by 0.7–3.4 times per unit increase in I/O ratio of temperature; decreased by 3%–6% per 1 °C increase in outdoor temperature
Humidity	C1-E, C2-E, and C2-L: Direction and statistical significance mixed within buildings, true effect unlikely	C1-E, C2-E, and C2-L: Direction and statistical significance mixed within buildings, true effect unlikely

Interestingly, the indoor PM mass concentration and I/O of PM in Building E during Campaign 1 were highest when the windows were open. We suspect that occupants were opening windows to air out the apartments, specifically to clear smoke or particles from other combustion sources; with a larger sample size we may have observed interaction effects of window opening and season like those previously observed in Pennsylvanian homes [28]. As was mentioned, this building is within 100 m of rail tracks and in an area known for a predominance of truck routes, both potential sources of airborne particles [52]. While measurements of infiltration were not available for either building, the strong contrasts in building construction and operation, and in observed PM levels, suggest that ventilation contributed to indoor air quality differences between the two buildings.

In addition, high concentrations of PM were more frequently measured in Building E during the second campaign (C2-E) than the first (C1-E). It is possible that different circumstances were encountered in the different years. Not only might some unmeasured building operational changes have occurred, but only 10 apartments were visited in both C1-E and C2-E; thus different occupants may have had different patterns of cooking, incense/candle burning, and smoking contributing to different PM levels between the two campaigns. The measurements made in this study were not meant to be representative of all apartments and times in these buildings, but rather to develop a general understanding of the concentrations typical to these apartments and the extent of consistency of those concentrations. All apartments in C2-E were scheduled within a 2-day period each month, and tenants had less flexibility with scheduling the C2-E visit than the C1-E visit. As a result, occupants may have modified their typical behavior more during C1-E than C2-E, leading to more frequent measurement during combustion in C2-E. In addition, during C1-E there were interventions conducted as part of a quasi-experimental project to promote healthy behaviors and improved IAQ. Although there may have been some time-dependence in the differences between these campaigns, the C2-E campaign repeated monitoring every month, so there does not appear to be a seasonal effect driving the differences between buildings, as would have happened if one campaign were conducted in summer and one in winter.

Finally, although we cannot rule out the possibility that some differences were related to different field staff, exactly the same instruments were calibrated by the same vendor and operated following manufacturer recommendations for all measurements.

### 4.2. Factors Affecting PM

We evaluated the effects of combustion-generating occupant behaviors, air conditioning, ventilation, and environmental conditions on PM. While pollutant sources could not be reliably identified, we observed several associations of occupant behavior and environmental conditions with PM mass concentrations in a single building (Table 3). For the most part, differences among apartments within a building were more evident in the frequency of high concentrations than in the central tendencies of concentrations. Combustion, particularly cooking with oil or burning incense, greatly increased PM mass concentration. While there was little effect of having a smoker live in an apartment on the medians of PM mass concentrations, PM mass concentrations >60 μg/m^3^ were 33% more likely for all size fractions measured in apartments where smokers lived than in non-smoking apartments in Building E. We expected smokers living in apartments to increase median PM mass concentrations like in previous studies [22,27]; this difference from other studies may have occurred because we only asked whether a smoker lived in the apartment, and not whether the smoker was often home or smoked in the apartment. The majority of the mass from cigarette smoke (typically 0.1–1.0 μm in diameter) [53] would have been captured by the DustTrak DRX since its sensitivity extends to 0.1 µm. Cigarette smoke would have contributed the most to PM_1_ and PM_2.5_ size fractions.

Other occupant behaviors like detailed information on time spent at home or frequency of consumer product use were not evaluated because the focus of this analysis was on behaviors related to combustion particles. We would expect similar background air pollutant levels in all apartments within a single building because the construction materials were the same, although differences due to the location of apartments within the buildings could not be ruled out. Unexplained between-apartment differences in PM may be due to occupant behaviors including cleaning and bringing dirt in from outside. In addition, some of the PM variability may be explained by the short measurement time in each apartment, which does not account for changes in smoking or AC use during the course of a day. 

The building ventilation and environmental conditions also affected indoor PM mass concentrations. Lower concentrations and I/O ratios were found for closed windows with or without AC. The effect of local exhaust ventilation on particle size fractions in Building E was small or not statistically significant. PM mass concentrations slightly decreased with increasing floor elevation in Building E (both campaigns) and C2-L; the smaller effect in C2-L may have been related to both the lack of sampling on lower floors and lower concentrations overall.

Sample size was a limitation when testing occupant behaviors that affect PM. In order to achieve reasonable power (*i.e.*, 0.8) where we saw small effects (e.g., window opening and AC operation) in Building E, samples of several hundred to thousands of measurements would be needed. Pooling all of the data from both buildings and campaigns could have feasibly resulted in sufficient power for comparisons; however, Building L had lower PM concentrations, higher ventilation rates, and less active occupants, all potentially confounding factors which could lead to incorrect interpretation of the pooled effects. Therefore, we are unable to report statistical significance for most factors tested, but can point at indications for future research in larger green building studies and recommend that in those studies all occupant behaviors that may affect PM concentrations (e.g., window opening and AC operation, combustion) be recorded in more detail. This will contribute to the literature on analyzing effects of occupant behaviors in green buildings on resident exposures to PM.

### 4.3. Comparison to Other Studies

Although suggestive, data from the collected literature do not provide sufficient evidence to conclude that green residential buildings have lower PM_2.5_ than traditional residential buildings. This is an important result in the context of increased construction of green buildings based on energy and environmental constraints that often do not prioritize health components of buildings. However, the number of studies was small and future studies may find differences, especially because variability in PM_2.5_ mass concentration was high across and within buildings in each study. Not all differences may be related to building design. Since green buildings may be newer than traditional buildings and some green building occupants may have self-selection bias, occupants of green buildings may more carefully control their indoor air quality, or at least have higher awareness about air quality issues. Because we compared ratios of PM_2.5_ calculated separately for each study, the variety of methods to measure PM_2.5_ (e.g., gravimetric, photometers) in different studies should not have affected our results. 

PM_2.5_ mass concentrations in our study were comparable to those in other studies in green residential buildings. In our measurements, we showed that the large amounts of variability between green buildings may be related to ambient PM mass concentrations and participant behaviors, including incense/candle burning, cooking on gas stoves (particularly with oil), and smoking. Therefore, PM mass concentrations measured in green residential buildings may sometimes be higher than PM mass concentrations in a given traditional building.

Additional measurements in buildings with a range of green building certifications are needed to determine whether these results can be generalized to other green residential buildings. The effects of occupant behavior and building design could be more clearly separated by including baseline measurements before the building is occupied, and then visiting occupied apartments with high-resolution measurement of PM as well as occupant behavior. These additional measurements would be used to guide interventions related to building design and occupant behavior to decrease PM concentrations in green residences, where people spend substantial and increasing amounts of time. These interventions could mirror existing occupant engagement and education in current green certifications (e.g., [4]).

## 5. Conclusions 

We demonstrated that two green high-rise residential buildings with differences in ventilation systems, operation, outdoor concentrations, and occupant behaviors have different profiles of PM mass concentrations and PM I/O. In one of these buildings, measurements made in different campaigns had different results, highlighting the need for sufficient repeated measurements and number of investigated apartments to characterize the PM levels in this building. In our study and others, PM_2.5_ varied both within and between green buildings. These findings highlight the importance of designing and operating green buildings to support health in addition to reducing environmental footprints. While increased resident health after moving into a green building has been demonstrated [17], the differences in PM concentrations among green buildings suggest that these improvements may not occur for all green buildings. For example, while air conditioner operation was associated with lower PM, participants with extremely low incomes who had window AC units and were responsible for their electric utility costs were often selective in when they ran the appliance. These participants lived in an area with some of the highest air pollution and asthma prevalence in the region. In addition, our data show that both building ventilation and occupant behaviors (e.g., use of active combustion sources in building) are important factors affecting residents’ exposure to PM in residential green buildings. Our results contribute to an emerging literature on PM in green buildings, and highlight the need to account for indoor air quality as well as energy and environmental impact when deciding whether a building should be considered green. Occupant education on air quality through behavioral interventions or other means could be adopted as an integral green building feature to improve air quality in the buildings.

## Supplementary Materials

The following are available online at www.mdpi.com/1660-4601/13/1/144/s1, Figure S1: Gantt chart of the measurement campaigns. Measurements were made in Building E in Campaign 1 (C1-E) Phases I, II, and III, as well as in Campaign 2 (C2-E). Measurements were made in Building L during Campaign 2 (C2-L). Figure S2: Histograms of particulate mass concentration (μg/m^3^) in Campaign 1 Building E (C1-E), and Campaign 2 Buildings E (C2-E) and L (C2-L) for (**a**) PM_1_; (**b**) PM_2.5_; (**c**) PM_4_; (**d**) PM_10_; and (**e**) PM_TOTAL_. Figure S3: Histograms of I/O in Campaign 1 Building E (C1-E; *n* = 55), Campaign 2 Building E (C2-E; *n* = 168), and Campaign 2 Building L (C2-L; *n* = 116) for (**a**) PM_1_; (**b**) PM_2.5_; (**c**) PM_4_; (**d**) PM_10_; and (**e**) PM_TOTAL_. Figure S4: Airborne particulate mass concentration (μg/m^3^) in Campaign 1 Building E (C1-E; *n* = 13) and Campaign 2 Building L (C2-L; 22 *n* = 116) apartments with closed windows and no active combustion for (**a**) PM_1_; (**b**) PM_2.5_; (**c**) PM_4_; (**d**) PM_10_; and (**e**) PM_TOTAL_. Different letters within the same box represent statistically significant (*p* < 0.05) groups under the Kruskal-Wallis multiple comparisons test. Figure S5: I/O in Campaign 1 Building E (C1-E; *n* = 13) and Campaign 2 Building L (C2-L; *n* = 116) apartments with closed windows and no active combustion for (**a**) PM_1_; (**b**) PM _2.5_; (**c**) PM_4_; (**d**) PM_10_; and (**e**) PM_TOTAL_. Different letters within the same box represent statistically 28 significant (*p* < 0.05) groups under the Kruskal-Wallis multiple comparisons test. Dashed lines for equal indoor and outdoor concentrations 29 (I/O = 1) are included for reference. Table S1: Summary statistics for PM size fractions measured in Building E during Campaigns 1 (C1-E), 2 (C2-E), and the pooled Campaigns (E), and in Building L during Campaign 2 (C2-L). Table S2: Log-linear regression relating PM mass concentration (μg/m^3^) and I/O to total exhaust ventilation rate in the kitchen and bathroom in Building E during Campaigns 1 (C1-E), 2 (C2-E), and both Campaigns (pooled E). Table S3: Temperature and relative humidity during sampling in Campaign 1 Building E (C1-E), Campaign 2 Building E (C2-E), and Campaign 2 Building L (C2-L). Table S4: Log-linear regression models for PM mass and I/O as a function of indoor and outdoor temperature, and the I/O ratio of temperature in Campaign 1 Building E (C1-E), Campaign 2 Building E (C2-E), the pooled data from Building E (pooled E), and Campaign 2 Building L (C2-L). Table S5: Log-linear regression models for PM mass and I/O as a function of indoor and outdoor relative humidity, and the I/O ratio of relative humidity in Campaign 1 Building E (C1-E), Campaign 2 Building E (C2-E), the pooled data from Building E (pooled E), and Campaign 2 Building L (C2-L). Statistically significant trends are in bold. Table S6: Kruskal-Wallis tests for particulate mass concentration (μg/m^3^) and indoor/outdoor ratio by building floor in Campaign 1 Building E (C1-E), Campaign 2 Building E (C2-E), the pooled data from Building E (E), and Campaign 2 Building L (C2-L). Table S7: Log-linear regression relating PM mass (μg/m^3^) and I/O to building floor number in Campaign 1 Building E (C1-E), Campaign 2 Building E (C2-E), the pooled data from Building E (pooled E), and Campaign 2 Building L (C2-L).
