# Supplementary material for: Airborne Particulate Matter in Two Multi-Family Green Buildings: Concentrations and Effect of Ventilation and Occupant Behavior
Source: Int J Environ Res Public Health. 2016 Jan 20;13(1):144. doi: 10.3390/ijerph13010144 (PMC4730535; doi:10.3390/ijerph13010144)
Supplement: Supplementary file 1 [file ijerph-13-00144-s001.pdf]

Int. J. Environ. Res. Public Health **2016**, *13*, 144; doi:10.3390/ijerph13010144

Supplementary Materials: Airborne Particulate Matter in Two Multi-Family Green Buildings: Concentrations and Effect of Ventilation and Occupant Behavior

Allison P. Patton, Leonardo Calderon, Youyou Xiong, Zuocheng Wang, Jennifer Senick, MaryAnn Sorensen Allacci, Deborah Plotnik, Richard Wener, Clinton J. Andrews, Uta Krogmann and Gediminas Mainelis

Table S1. Summary statistics for PM size fractions measured in Building E during Campaigns 1 (C1-E), 2 (C2-E), and the pooled Campaigns (E), and in Building L during Campaign 2 (C2-L).

Measure	Statistic [a]	Mass Concentration μg/m³				Indoor/Outdoor Ratio			
		C1-E	C2-E	E	C2-L	C1-E	C2-E	E	C2-L
PM_1	n	55	168	223	116	55	168	223	116
	min	8	7	7	2	0.52	0.24	0.24	0.06
	Q1	18	23	22	13	0.98	0.92	0.92	0.28
	median	26	38	33	18	1.42	1.31	1.32	0.49
	mean	34	88	74	23	2.02	4.76	4.08	0.63
	Q3	40	70	62	25	1.89	3.08	2.76	0.73
	max	138	862	862	134	8.92	66.31	66.31	6.25
$PM_{2.5}$	min	8	7	7	2	0.53	0.25	0.25	0.06
	Q1	19	24	22	13	0.99	0.93	0.94	0.28
	median	27	38	34	18	1.41	1.28	1.32	0.48
	mean	34	89	75	23	2.03	4.74	4.07	0.63
	max	138	862	862	135	9.26	66.31	66.31	6.33
PM_4	min	9	7	7	2	0.53	0.24	0.24	0.07
	Q1	20	25	23	14	0.99	0.93	0.95	0.28
	median	28	40	34	20	1.44	1.3	1.33	0.5
	mean	36	90	76	24	2.07	4.74	4.08	0.64
	Q3	42	74	64	27	1.92	3.09	2.84	0.75
	max	139	862	862	136	9.64	66.31	66.31	6.42
PM_{10}	min	12	8	8	2	0.56	0.26	0.26	0.08
	Q1	23	27	26	18	1.02	1	1	0.29
	median	33	42	39	23	1.52	1.4	1.4	0.54
	mean	41	93	80	28	2.19	4.76	4.13	0.69
	Q3	47	77	67	33	2.1	3.05	2.7	0.87
	max	142	864	864	137	9.57	61.71	61.71	6.15
PM_{TOTAL}	min	20	14	14	2	0.72	0.4	0.4	0.12
	Q1	36	38	36	28	1.21	1.25	1.24	0.35
	median	52	59	56	37	2.06	1.93	1.96	0.78
	mean	56	109	96	41	2.73	4.99	4.44	0.97
	Q3	63	97	82	49	3.28	4.29	3.62	1.25
	max	156	873	873	141	11.45	58.2	58.2	6.14

[a] The statistics reported are number of apartment visits (n), minimum (min), first quartile (Q1), median, mean, third quartile (Q3), and maximum (max).

Table S2. Log-linear regression relating PM mass concentration (μg/m^3) and I/O to total exhaust ventilation rate in the kitchen and bathroom in Building E during Campaigns 1 (C1-E), 2 (C2-E), and both Campaigns (pooled E).

Site	Pollutant	Mass Concentration (μg/m^3)			I/O		
		Estimate	Standard Error	*p*-Value	Estimate	Standard Error	*p*-Value
C1-E	PM_1	0.002	0.003	0.64	-0.002	0.003	0.54
	$PM_{2.5}$	0.002	0.003	0.64	−0.002	0.003	0.53
	PM_4	0.002	0.003	0.64	−0.002	0.003	0.52
	PM_{10}	0.002	0.003	0.62	−0.002	0.003	0.51
	PM_{TOTAL}	0.001	0.003	0.76	−0.004	0.003	0.30
C2-E	PM_1	−0.003	0.003	0.19	−0.003	0.003	0.26
	$PM_{2.5}$	−0.003	0.003	0.19	−0.003	0.003	0.27
	PM_4	−0.003	0.003	0.19	−0.003	0.003	0.26
	PM_{10}	−0.003	0.003	0.17	−0.003	0.003	0.24
	PM_{TOTAL}	−0.004	0.002	0.04	−0.004	0.003	0.11
Pooled E	PM_1	−0.003	0.002	0.19	−0.003	0.002	0.19
	$PM_{2.5}$	−0.003	0.002	0.20	−0.003	0.002	0.20
	PM_4	−0.003	0.002	0.19	−0.003	0.002	0.19
	PM_{10}	−0.003	0.002	0.18	−0.003	0.002	0.17
	PM_{TOTAL}	−0.004	0.002	0.05	−0.004	0.002	0.06

Table S3. Temperature and relative humidity during sampling in Campaign 1 Building E (C1-E), Campaign 2 Building E (C2-E), and Campaign 2 Building L (C2-L).

Measure	Statistic [a]	Indoor			Outdoor		
		C1-E	C2-E	C2-L	C1-E	C2-E	C2-L
Temperature, °F (°C)	n	55	168	116	55	168	85
	n missing	0	0	0	0	0	31
	min	69 (20.6)	67 (19.4)	68 (20)	48 (8.9)	20 (-6.7)	47 (8.3)
	Q1	76 (24.4)	76 (24.4)	72 (22.2)	56 (13.3)	36 (2.2)	67 (19.4
	median	77 (25)	78 (25.6)	74 (23.3)	66 (18.9)	57 (13.9)	70 (21.1)
	mean	78 (25.6)	78 (25.6)	74 (23.3)	68 (20)	58 (14.4)	70 (21.1)
	Q3	81 (27.2)	81 (27.2)	75 (23.9)	79 (26.1)	80 (26.7)	78 (25.6)
	max	87 (30.6)	89 (31.7)	80 (26.7)	94 (34.4)	90 (32.2)	79 (26.1)
Relative Humidity, %	n	55	168	116	55	168	93
	n missing	0	0	0	0	0	23
	min	13	6	26	22	24	44
	Q1	33	21	50	38	39	50
	median	41	40	54	44	48	69
	mean	40	38	53	47	48	66
	Q3	46	54	59	49	60	78
	max	68	71	71	74	71	87

[a] The statistics reported are number of apartment visits (n), minimum (min), first quartile (Q1), median, mean, third quartile (Q3), and maximum (max).

Table S4. Log-linear regression models for PM mass and I/O as a function of indoor and outdoor temperature, and the I/O ratio of temperature in Campaign 1 Building E (C1-E), Campaign 2 Building E (C2-E), the pooled data from Building E (pooled E), and Campaign 2 Building L (C2-L).

Site	Pollutant	Mass Concentration (μg/m³)			I/O		
		Estimate	Standard Error	*p*-Value	Estimate	Standard Error	*p*-Value
Indoor Temperature, °C							
C1-E	PM_1	0.01	0.04	0.88	−0.02	0.04	0.69
	$PM_{2.5}$	0.01	0.04	0.88	−0.02	0.04	0.66
	PM_4	0.004	0.04	0.91	−0.02	0.04	0.63
	PM_{10}	−0.003	0.03	0.93	−0.03	0.04	0.49
	PM_{TOTAL}	−0.02	0.03	0.59	−0.05	0.04	0.23
C2-E	PM_1	0.04	0.04	0.26	−0.07	0.04	0.08
	$PM_{2.5}$	0.04	0.04	0.27	−0.07	0.04	0.08
	PM_4	0.04	0.04	0.29	−0.07	0.04	0.07
	PM_{10}	0.03	0.03	0.34	−0.07	0.04	0.05
	PM_{TOTAL}	0.02	0.03	0.55	−0.09	0.03	0.01
Pooled E	PM_1	0.04	0.03	0.22	−0.05	0.03	0.09
	$PM_{2.5}$	0.04	0.03	0.23	−0.05	0.03	0.08
	PM_4	0.03	0.03	0.24	−0.06	0.03	0.07
	PM_{10}	0.03	0.03	0.33	−0.06	0.03	0.04
	PM_{TOTAL}	0.01	0.02	0.60	−0.08	0.03	0.01
C2-L	PM_1	0.01	0.05	0.89	−0.001	0.06	0.99
	$PM_{2.5}$	0.003	0.05	0.94	−0.004	0.06	0.96
	PM_4	0.001	0.05	0.98	−0.01	0.06	0.91
	PM_{10}	0.01	0.04	0.90	−0.01	0.06	0.93
	PM_{TOTAL}	0.01	0.04	0.80	−0.002	0.07	0.97
Indoor/Outdoor ratio of Temperature, (°C/°C)							
C1-E	PM_1	0.21	0.71	0.77	0.65	0.81	0.43
	$PM_{2.5}$	0.21	0.71	0.77	0.66	0.82	0.42
	PM_4	0.22	0.70	0.76	0.69	0.82	0.41
	PM_{10}	0.30	0.66	0.65	0.76	0.80	0.35
	PM_{TOTAL}	0.50	0.55	0.37	1.06	0.80	0.20
C2-E	PM_1	−0.23	0.20	0.23	0.71	0.20	0.001
	$PM_{2.5}$	−0.23	0.19	0.23	0.72	0.20	0.001
	PM_4	−0.23	0.19	0.24	0.72	0.20	0.001
	PM_{10}	−0.20	0.19	0.29	0.78	0.20	0.0001
	PM_{TOTAL}	−0.10	0.16	0.52	0.83	0.18	<0.0001
Pooled E	PM_1	−0.06	0.17	0.74	0.72	0.18	0.0001
	$PM_{2.5}$	−0.06	0.17	0.74	0.73	0.18	<0.0001
	PM_4	−0.06	0.17	0.72	0.72	0.18	0.0001
	PM_{10}	−0.06	0.16	0.72	0.77	0.17	<0.0001
	PM_{TOTAL}	0.01	0.14	0.92	0.83	0.16	<0.0001
C2-L	PM_1	−0.12	0.68	0.86	2.23	0.83	0.01
	$PM_{2.5}$	−0.11	0.68	0.87	2.23	0.83	0.01
	PM_4	−0.03	0.66	0.96	2.44	0.81	0.003
	PM_{10}	0.19	0.61	0.75	2.89	0.81	0.001
	PM_{TOTAL}	0.54	0.53	0.31	3.48	0.86	0.0001
Outdoor Temperature, °C							
C1-E	PM_1	0.001	0.01	0.93	−0.01	0.01	0.38
	$PM_{2.5}$	0.001	0.01	0.94	−0.01	0.01	0.37
	PM_4	0.0004	0.01	0.97	−0.01	0.01	0.35
	PM_{10}	−0.002	0.01	0.85	−0.01	0.01	0.26
	PM_{TOTAL}	−0.01	0.01	0.44	−0.02	0.01	0.10

Table S4. *Cont.*

Site	Pollutant	Mass Concentration (μg/m³)			I/O		
		Estimate	Standard Error	*p*-Value	Estimate	Standard Error	*p*-Value
		Outdoor Temperature, °C					
C2-E	PM_1	0.01	0.01	0.27	−0.03	0.01	0.0001
	$PM_{2.5}$	0.01	0.01	0.27	−0.03	0.01	0.0001
	PM_4	0.01	0.01	0.28	−0.03	0.01	0.0001
	PM_{10}	0.01	0.01	0.36	−0.03	0.01	<0.0001
	PM_{TOTAL}	0.002	0.01	0.77	−0.03	0.01	<0.0001
Pooled E	PM_1	0.003	0.01	0.64	−0.03	0.01	<0.0001
	$PM_{2.5}$	0.003	0.01	0.65	−0.03	0.01	<0.0001
	PM_4	0.003	0.01	0.65	−0.03	0.01	<0.0001
	PM_{10}	0.002	0.01	0.72	−0.03	0.01	<0.0001
	PM_{TOTAL}	−0.002	0.005	0.71	−0.03	0.01	<0.0001
C2-L	PM_1	0.005	0.01	0.68	−0.04	0.01	0.01
	$PM_{2.5}$	0.005	0.01	0.71	−0.04	0.01	0.01
	PM_4	0.003	0.01	0.79	−0.04	0.01	0.01
	PM_{10}	−0.001	0.01	0.96	−0.05	0.01	0.001
	PM_{TOTAL}	−0.01	0.01	0.54	−0.06	0.02	0.0003

Table S5. Log-linear regression models for PM mass and I/O as a function of indoor and outdoor relative humidity, and the I/O ratio of relative humidity in Campaign 1 Building E (C1-E), Campaign 2 Building E (C2-E), the pooled data from Building E (pooled E), and Campaign 2 Building L (C2-L). Statistically significant trends are in bold.

Site	Pollutant	Mass Concentration (μg/m³)			I/O		
		Estimate	Standard Error	*p*-Value	Estimate	Standard Error	*p*-Value
		Indoor Relative Humidity, %					
C1-E	PM_1	0.008	0.007	0.297	0.007	0.008	0.398
	$PM_{2.5}$	0.008	0.007	0.287	0.007	0.008	0.417
	PM_4	0.007	0.007	0.299	0.006	0.008	0.445
	PM_{10}	0.006	0.007	0.387	0.006	0.008	0.497
	PM_{TOTAL}	0.003	0.006	0.633	0.003	0.008	0.683
C2-E	PM_1	0.010	0.005	0.026	−0.015	0.005	**0.003**
	$PM_{2.5}$	0.010	0.005	**0.027**	−0.015	0.005	**0.002**
	PM_4	0.010	0.005	**0.026**	−0.015	0.005	**0.002**
	PM_{10}	0.009	0.004	**0.033**	−0.016	0.005	**<0.001**
	PM_{TOTAL}	0.005	0.004	0.152	−0.020	0.004	**<0.001**
Pooled E	PM_1	0.009	0.004	**0.023**	−0.012	0.004	**0.003**
	$PM_{2.5}$	0.009	0.004	**0.023**	−0.013	0.004	**0.003**
	PM_4	0.009	0.004	**0.022**	−0.013	0.004	**0.002**
	PM_{10}	0.008	0.004	**0.028**	−0.014	0.004	**<0.001**
	PM_{TOTAL}	0.005	0.003	0.156	−0.017	0.004	**<0.001**
C2-L	PM_1	0.017	0.006	**0.004**	−0.012	0.007	0.114
	$PM_{2.5}$	0.017	0.006	**0.004**	−0.012	0.007	0.109
	PM_4	0.016	0.006	**0.006**	−0.013	0.007	**0.066**
	PM_{10}	0.013	0.006	0.021	−0.017	0.007	**0.021**
	PM_{TOTAL}	0.008	0.005	0.153	−0.024	0.008	**0.002**

Table S5. *Cont.*

Site	Pollutant	Mass Concentration (μg/m³)			I/O		
		Estimate	Standard Error	*p*-Value	Estimate	Standard Error	*p*-Value
Indoor/Outdoor ratio of Relative Humidity, %							
C1-E	PM_1	0.40	0.34	0.24	0.11	0.40	0.78
	$PM_{2.5}$	0.41	0.34	0.24	0.12	0.40	0.76
	PM_4	0.40	0.34	0.24	0.12	0.40	0.76
	PM_{10}	0.34	0.32	0.29	0.06	0.40	0.88
Indoor/Outdoor ratio of Relative Humidity, %							
	PM_{TOTAL}	0.20	0.27	0.47	−0.16	0.40	0.68
C2-E	PM_1	0.42	0.23	0.06	−0.30	0.24	0.22
	$PM_{2.5}$	0.42	0.23	0.07	−0.30	0.24	0.21
	PM_4	0.41	0.23	0.07	−0.31	0.24	0.20
	PM_{10}	0.38	0.22	0.08	−0.36	0.24	0.13
	PM_{TOTAL}	0.25	0.19	0.18	−0.46	0.22	**0.03**
Pooled E	PM_1	0.32	0.20	0.10	−0.29	0.21	0.17
	$PM_{2.5}$	0.32	0.20	0.10	−0.29	0.21	0.17
	PM_4	0.32	0.19	0.10	−0.29	0.21	0.16
	PM_{10}	0.30	0.19	0.11	−0.34	0.20	0.10
	PM_{TOTAL}	0.18	0.16	0.26	−0.45	0.19	**0.02**
C2-L	PM_1	−0.87	0.37	**0.02**	1.76	0.43	**<0.001**
	$PM_{2.5}$	−0.87	0.37	**0.02**	1.79	0.43	**<0.001**
	PM_4	−0.87	0.36	**0.02**	1.67	0.43	**<0.001**
	PM_{10}	−0.84	0.33	**0.01**	1.39	0.45	**0.00**
	PM_{TOTAL}	−0.66	0.28	**0.02**	1.28	0.49	**0.01**
Outdoor Relative Humidity, %							
C1-E	PM_1	0.0001	0.006	0.99	0.004	0.007	0.61
	$PM_{2.5}$	0.0002	0.006	0.97	0.003	0.007	0.65
	PM_4	0.0001	0.006	0.99	0.003	0.007	0.67
	PM_{10}	−0.0003	0.006	0.95	0.003	0.007	0.62
	PM_{TOTAL}	−0.0004	0.005	0.94	0.006	0.007	0.43
C2-E	PM_1	0.010	0.006	0.09	−0.016	0.006	**0.02**
	$PM_{2.5}$	0.010	0.006	0.09	−0.016	0.006	**0.01**
	PM_4	0.010	0.006	0.08	−0.016	0.006	**0.01**
	PM_{10}	0.010	0.006	0.09	−0.017	0.006	**0.01**
	PM_{TOTAL}	0.006	0.005	0.22	−0.020	0.006	**<0.001**
Pooled E	PM_1	0.009	0.005	0.08	−0.011	0.005	**0.04**
	$PM_{2.5}$	0.009	0.005	0.08	−0.011	0.005	**0.03**
	PM_4	0.009	0.005	0.07	−0.011	0.005	**0.03**
	PM_{10}	0.008	0.005	0.08	−0.012	0.005	**0.02**
	PM_{TOTAL}	0.005	0.004	0.20	−0.014	0.005	**0.003**
C2-L	PM_1	0.018	0.004	**<0.001**	−0.023	0.005	**<0.001**
	$PM_{2.5}$	0.018	0.004	**<0.001**	−0.023	0.005	**<0.001**
	PM_4	0.017	0.004	**<0.001**	−0.023	0.005	**<0.001**
	PM_{10}	0.015	0.003	**<0.001**	−0.023	0.005	**<0.001**
	PM_{TOTAL}	0.009	0.003	**0.003**	−0.026	0.005	**<0.001**

Table S6. Kruskal-Wallis tests for particulate mass concentration (μg/m^3) and indoor/outdoor ratio by building floor in Campaign 1 Building E (C1-E), Campaign 2 Building E (C2-E), the pooled data from Building E (E), and Campaign 2 Building L (C2-L).

Site	Pollutant	*p*-Value	
		Mass Concentration	Indoor/Outdoor Ratio
C1-E	PM_1	0.114	0.363
	$PM_{2.5}$	0.117	0.322
	PM_4	0.09	0.289
	PM_{10}	0.063	0.321
	PM_{TOTAL}	0.031	0.421
C2-E	PM_1	0.008	0.022
	$PM_{2.5}$	0.008	0.019
	PM_4	0.009	0.027
	PM_{10}	0.015	0.049
	PM_{TOTAL}	0.024	0.255
E	PM_1	0.001	0.009
	$PM_{2.5}$	0.001	0.007
	PM_4	0.002	0.011
	PM_{10}	0.003	0.027
	PM_{TOTAL}	0.008	0.209
C2-L	PM_1	0.078	0.501
	$PM_{2.5}$	0.071	0.504
	PM_4	0.077	0.479
	PM_{10}	0.103	0.604
	PM_{TOTAL}	0.043	0.678

Table S7. Log-linear regression relating PM mass (μg/m^3) and I/O to building floor number in Campaign 1 Building E (C1-E), Campaign 2 Building E (C2-E), the pooled data from Building E (pooled E), and Campaign 2 Building L (C2-L).

Site	Pollutant	Mass Concentration (μg/m^3)			I/O		
		Estimate	Standard Error	p-Value	Estimate	Standard Error	p-Value
C2-E	PM_1	−0.07	0.04	0.13	−0.05	0.05	0.33
	$PM_{2.5}$	−0.07	0.04	0.14	−0.05	0.05	0.34
	PM_4	−0.07	0.04	0.14	−0.05	0.05	0.35
	PM_{10}	−0.07	0.04	0.12	−0.05	0.05	0.33
	PM_{TOTAL}	−0.06	0.04	0.11	−0.04	0.04	0.39
C1-E	PM_1	−0.05	0.04	0.19	−0.08	0.04	0.05
	$PM_{2.5}$	−0.05	0.04	0.18	−0.08	0.04	0.05
	PM_4	0.05	0.04	0.17	−0.08	0.04	0.05
	PM_{10}	−0.05	0.03	0.14	−0.09	0.04	0.04
	PM_{TOTAL}	−0.04	0.03	0.13	−0.09	0.04	0.04
Pooled E	PM_1	−0.07	0.03	0.06	−0.06	0.04	0.10
	$PM_{2.5}$	−0.07	0.03	0.06	−0.06	0.04	0.10
	PM_4	−0.07	0.03	0.05	−0.06	0.04	0.10
	PM_{10}	−0.06	0.03	0.05	−0.06	0.04	0.09
	PM_{TOTAL}	−0.06	0.03	0.04	−0.06	0.03	0.09
C2-L	PM_1	−0.01	0.01	0.31	−0.02	0.01	0.29
	$PM_{2.5}$	−0.01	0.01	0.32	−0.01	0.01	0.31
	PM_4	−0.01	0.01	0.29	−0.02	0.01	0.28
	PM_{10}	−0.01	0.01	0.19	-0.02	0.01	0.24
	PM_{TOTAL}	−0.02	0.01	0.03	−0.02	0.02	0.13

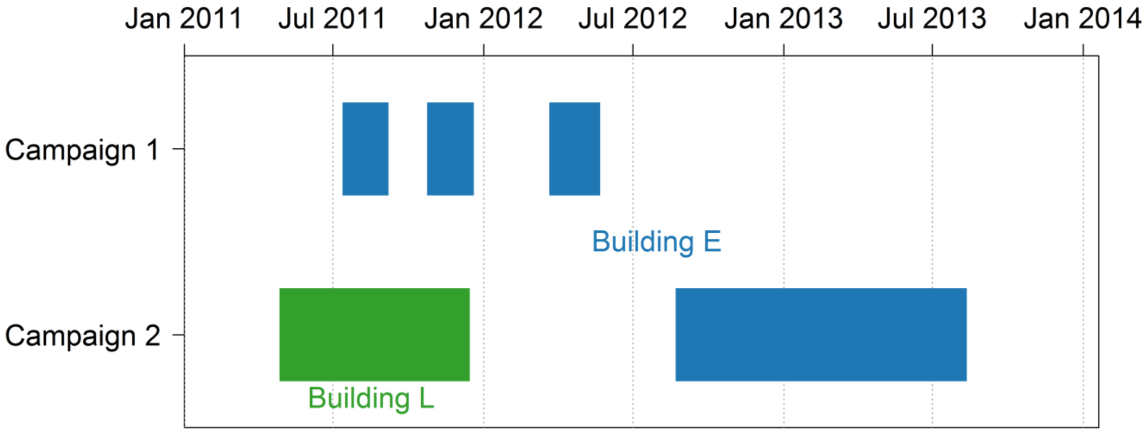

Figure S1. Gantt chart of the measurement campaigns. Measurements were made in Building E in Campaign 1 (C1-E) Phases I, II, and III, as well as in Campaign 2 (C2-E). Measurements were made in Building L during Campaign 2 (C2-L).

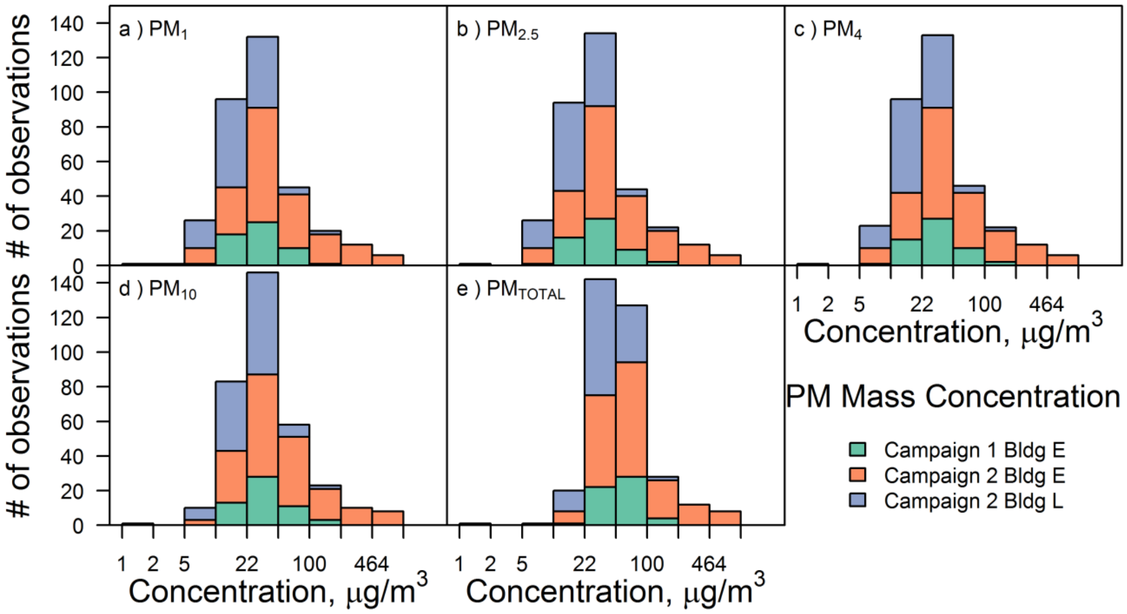

Figure S2. Histograms of particulate mass concentration (µg/m³) in Campaign 1 Building E (C1-E), and Campaign 2 Buildings E (C2-E) and L (C2-L) for (**a**) PM_1; (**b**) $PM_{2.5}$; (**c**) PM_4; (**d**) PM_{10}; and (**e**) PM_{TOTAL}.

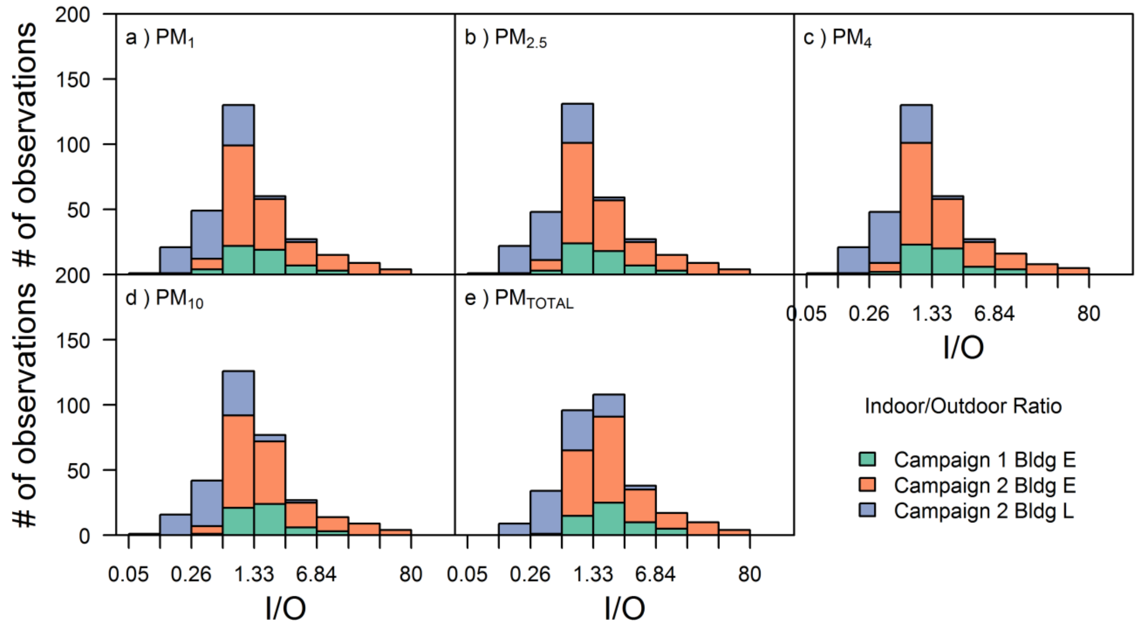

Figure S3. Histograms of I/O in Campaign 1 Building E (C1-E; $n = 55$), Campaign 2 Building E (C2-E; $n = 168$), and Campaign 2 Building L (C2-L; $n = 116$) for (**a**) PM_1; (**b**) $PM_{2.5}$; (**c**) PM_4; (**d**) PM_{10}; and (**e**) PM_{TOTAL}.

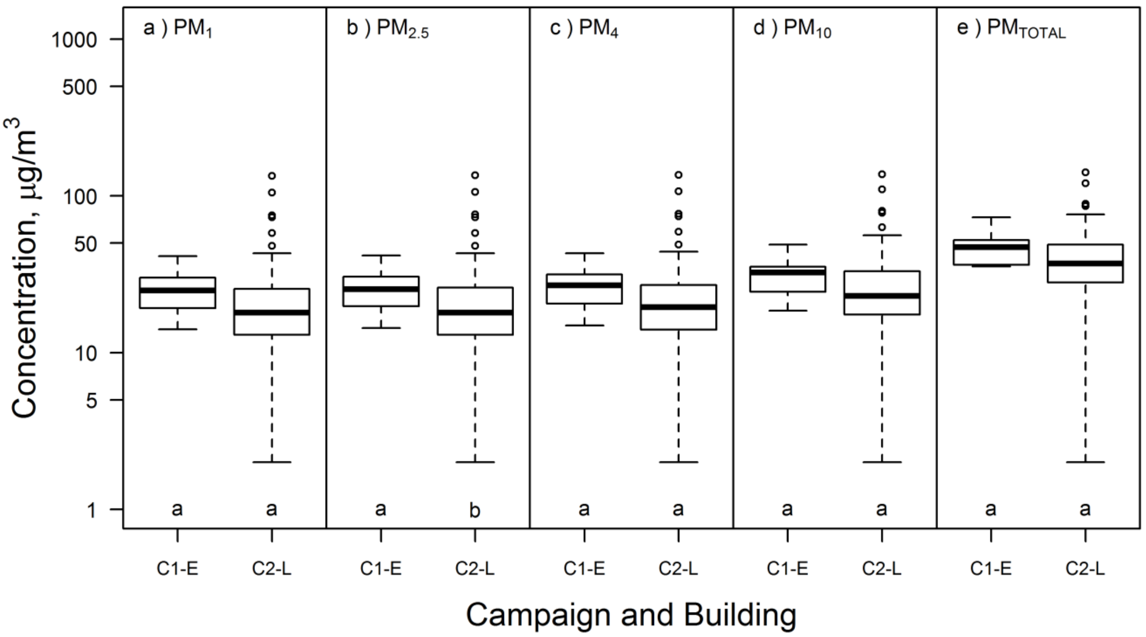

Figure S4. Airborne particulate mass concentration (μg/m³) in Campaign 1 Building E (C1-E; $n = 13$) and Campaign 2 Building L (C2-L; 22 $n = 116$) apartments with closed windows and no active combustion for (**a**) PM_1; (**b**) $PM_{2.5}$; (**c**) PM_4; (**d**) PM_{10}; and (**e**) PM_{TOTAL}. Different letters within the same box represent statistically significant ($p < 0.05$) groups under the Kruskal-Wallis multiple comparisons test.

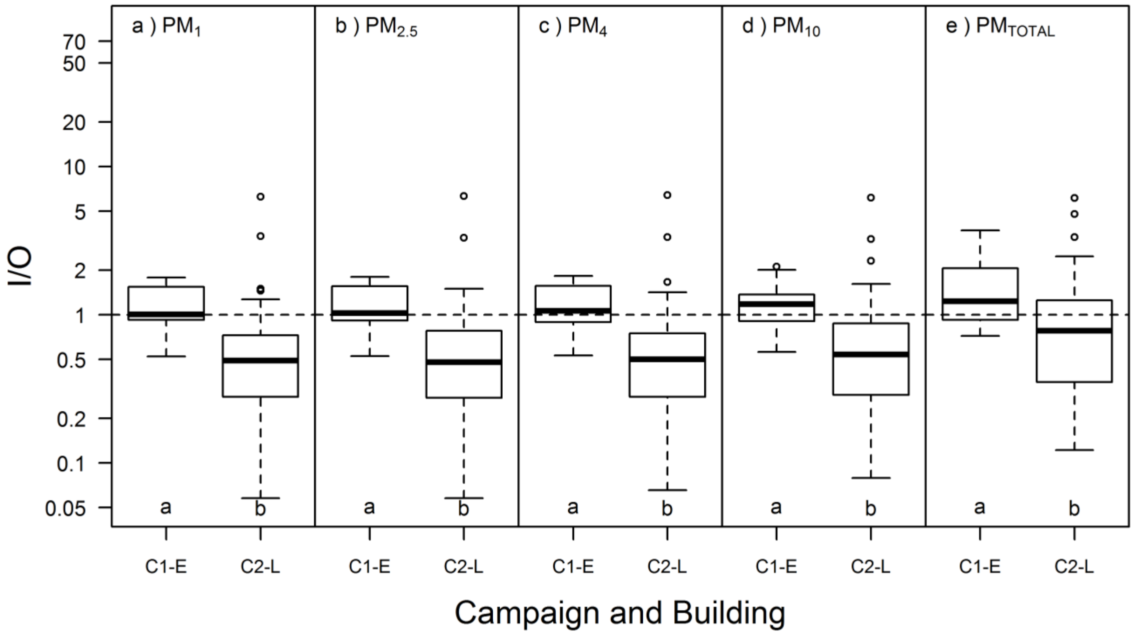

Figure S5. I/O in Campaign 1 Building E (C1-E; n = 13) and Campaign 2 Building L (C2-L; n = 116) apartments with closed windows and no active combustion for (**a**) PM_1; (**b**) $PM_{2.5}$; (**c**) PM_4; (**d**) PM_{10}; and (**e**) PM_{TOTAL}. Different letters within the same box represent statistically 28 significant ($p < 0.05$) groups under the Kruskal-Wallis multiple comparisons test. Dashed lines for equal indoor and outdoor concentrations 29 (I/O = 1) are included for reference.

© 2016 by the authors; licensee MDPI, Basel, Switzerland. This article is an open access article distributed under the terms and conditions of the Creative Commons by Attribution (CC-BY) license (http://creativecommons.org/licenses/by/4.0/).
